# A new model for health care delivery

**DOI:** 10.1186/1472-6963-9-57

**Published:** 2009-04-01

**Authors:** John P Kepros, Razvan C Opreanu

**Affiliations:** 1 Department of Surgery, Michigan State University, East Lansing, MI, USA; 2 Sparrow Health System, Lansing, MI, USA

## Abstract

**Background:**

The health care delivery system in the United States is facing cost and quality pressures that will require fundamental changes to remain viable. The optimal structures of the relationships between the hospital, medical school, and physicians have not been determined but are likely to have a large impact on the future of healthcare delivery. Because it is generally agreed that academic medical centers will play a role in the sustainability of this future system, a fundamental understanding of the relative contributions of the stakeholders is important as well as creativity in developing novel strategies to achieve a shared vision.

**Discussion:**

Core competencies of each of the stakeholders (the hospital, the medical school and the physicians) must complement the others and should act synergistically. At the same time, the stakeholders should determine the common core values and should be able to make a meaningful contribution to the delivery of health care.

**Summary:**

Health care needs to achieve higher quality and lower cost. Therefore, in order for physicians, medical schools, and hospitals to serve the needs of society in a gratifying way, there will need to be change. There needs to be more scientific and social advances. It is obvious that there is a real and urgent need for relationship building among the professionals whose duty it is to provide these services.

## Background

### The Need for Change

Currently the United States' health care system is facing pressure with respect to quality and cost. Despite being the most expensive system in the world in terms of the share of gross domestic product [1], the life expectancy from birth is one of the lowest of the developed countries [2]. Despite the lack of a definitive resolution from the myriad discussions of the topic, it is generally understood that academic centers will play a role in the eventual solution [3-6].

Historically, despite several high profile exceptions, the relationship between hospitals and universities has been, at times, a strained one and many academic medical centers report concerns about their viability [3,6-12]. Many successful medical centers are also independent of universities and many of the innovations in medicine have come from private practice [13-16]. For example, laparoscopic gallbladder removal, a common procedure in modern health care, was pioneered by private physicians [17]. Managed care was also created out of a commercial need for health care. For example, Kaiser Permanente was created to fill the needs of Industrial Indemnity as a way to provide prepayment for industrial healthcare [18]. Furthermore, in terms of quality, private hospitals have shown more recent success in adopting industry level initiatives for improving care [19-26]. Because the role of education and research are fundamentally critical for the sustainability of the health care system, however, a new, more creative, model must be formulated from the complex array of pressures and influences that characterize the current health care system. Fortunately there is significant literature regarding both the health care delivery and organizational building aspects of this challenge. In particular, there has been relevant research in the last several decades about using a core ideology for organization building and leadership [27-31]. This paper will emphasize a vision that includes, not only the structure of this new model which should incorporate quality, customer service, innovation, and sustainability, but also the urgency with which this system is needed.

### Historical Aspects of Hospital and University Relationships

It is often assumed that hospital and university alliances have remained constant. A historical review, however, reveals that this is a constantly evolving relationship. Massachusetts General Hospital (MGH), one of the oldest and best-known teaching hospitals in the United States, has a well-known affiliation with Harvard University but is actually currently owned by Partners Healthcare™, a non-profit organization [32]. It was not until 1869, 58 years after the founding of MGH in 1811, that the University of Michigan opened the first university-owned medical facility in the United States [33]. Yale-New Haven Hospital did not have "Yale" in its title at all until 1965 and is currently owned by the Yale-New Haven Health System Inc [34]. Regardless of the nature of the ownership, most health care delivery systems are more closely associated with the hospital than a university. For example, the Centers for Medicare and Medicaid Services (CMS) through the Medicare Part A trust fund, give funding to hospitals for graduate medical education [35]. The Joint Commission accredits hospitals based on a set of standards [36]. Trauma verification is given to individual hospitals, not to health systems or universities [37]. The quality awards mentioned above are typically given to hospitals. Furthermore, because this association is so strong, the identity of the hospital becomes critical for marketing purposes [38]. Finally, with declining physician reimbursement from Medicare, hospital subsidizing will likely play a larger role in some physician salaries [39]. Universities, however, have expertise in research, education, and leadership that are not available elsewhere.

## Discussion

### Sustainability: Education, Research, The Uninsured and Succession Planning for Leadership

Despite the compelling reactive urge for hospitals to identify an immediate solution to the cost and quality crisis, the need to proactively and concurrently develop a long-term solution is as important and as urgent. There is evidence of a physician shortage [40-42], a critical need for relevant research [43,44], a need to care for the large number of uninsured people in the United States [45], and a critical need for effective, durable leadership in medicine [31]. These exigent needs cannot be overlooked, play a significant role in our current crisis and will need to be woven into the fabric of the solution alluded to above. The physician shortage will be exacerbated without new physicians to replace retiring physicians. There must also be innovation and the spread of new ideas to maintain progress in the profession of medicine. Physicians are also expected to be leaders in the field. They are most knowledgeable about diagnosis and treatment as well as the health care system. Thus, the competencies of a university and the university involvement are critical to the sustainability of any system of health care.

### Essential Stakeholders: Patients, the Hospital, the Medical School and the Physicians

The interface of any health care system is the patient experience with providers. As with other consumer experiences, the consumer (patient) expects a organized, coordinated effort within the organization to make this experience pleasant and effective. To provide this patient-centered care, the hospital, medical school, and physicians should have a shared vision and common understanding of the goals to direct their activity. A key concept involved in any collaborative effort is synergy. More specifically, the core competencies of each of the stakeholders must complement the others [46]. In the case of health care, the hospital, medical school, and physicians all bring core competencies to the collaboration that should act synergistically. The hospital brings the identity (mentioned above), the physical plant, and funding sources. The medical school brings with it the crucial sustainability components of education and research. These components should not be underestimated in light of the current physician shortage and increasing need for evidence based practices [47]. There are also frequently divisional needs for research such as for trauma center verification by the American College of Surgeons [37]. The core competencies of the physicians as a whole are their role in the workforce, patient satisfaction, and hopefully leadership. The precise nature of medical leadership is not completely known as it is responding to challenges from the changes in the healthcare environment [48].

### Toward a Shared Vision

Adherence to long-held traditions in medicine in the face of the jolting facts of reality regarding cost and quality has left the medical literature with several examples of successful models but no underlying principles or unifying theories [49-57]. In essence this means that there is justification for building a model from scratch, drawing upon successful models for elements of theory. Besides weaving in the long-term sustainability thread mentioned above, there is also a unique opportunity to build a shared vision between the stakeholders that could not be done in a time of more stability.

It is also fortunate that there is guidance from research on organizational structure. Two sets of comparison studies [27,58] and an expansion of the lessons learned into the structure of social organizations such as hospitals and universities [28] that provide the essential but sometimes counterintuitive basis for successful organization development are examples. In *Built to Last: Successful Habits of Visionary Companies*, Jim Collins and Jerry Porras surveyed CEO's of major organizations to select longstanding Fortune 500 companies that were considered visionary and compared these to a control group that were less successful in an effort to identify characteristics of these successful companies that could possibly be adopted by other organizations. The remaining question, which Jim Collins answered in *Good to Great: Why Some Companies Make the Leap...and Others Don't *was how an organization could go from mediocrity to excellence if it was not always that way. He compared companies with relatively flat earnings over a 15 year period that then had an increase in earnings for another 15 year period to very similar companies whose earnings remained flat. Both of these studies were modeled after twin studies. Of the concepts presented in this collection, a fundamental one is the principle of preserving the core ideology. The core ideology is presented as a combination of the core values, which are the values the organization holds above all others and would hold even if it meant the organization would go out of business and the core purpose which is the reason the organization exists and which should be valid for the next 100 years [58]. For example, 3 M states its purpose is to produce imaginative products and values employee talent and initiative. If the core ideology remains stable then it is easier to change anything thing else that needs changing. One of the first steps then, is to determine the core ideology of an organization and then take a very critical look at everything else holding nothing sacred. Although it may take some discussion, the stakeholders (hospital, medical school, physicians) should determine the common core values and then recognize and accept that other, previously held beliefs may have to be dismissed.

### A Proposal for Meaningful Relationship Building

This new model of healthcare should represent a organized and integrated effort to create a system of care that optimally utilizes all of its components and represents patients as the primary stakeholders. This is in contrast to the current system in which patients, medical colleges, physicians, and hospitals all have, to a large extent, separate goals. The measure of the success of any system should be the impact on quality and cost. As mentioned above, life expectancy from birth and the share of gross domestic product are important measures.

A common core ideology of all of the stakeholders would likely include measurable improvements in patient-centered outcomes as well as improvements in public health. Each stakeholder, based on its core competencies, should be able to make a meaningful and gratifying contribution to this shared vision and purpose. The hospital's core competencies include the facility with facility funding, identity, institutional quality improvement processes, and strategic planning as it relates to organizational growth. As mentioned above hospitals commonly have identities distinct from medical schools and physicians although the relationships with these groups are essential.

The medical school's core competencies may include intellectual expertise in research that has the potential to raise the profile and reputation of the entire healthcare organization and the commitment to teaching another generation of professionals. Both of these are absolute necessities for sustainability. The medical school should, however have a clear recognition of its purpose. Jim Collins, in an address at the 2006 American Association of Medical Colleges (AAMC) annual meeting suggested, "social sector organizations like the AAMC and academic medical centers should avoid thinking like businesses because financial performance is not a measure of success". "To thrive in an environment of high expectations and dwindling resources", Collins said, "academic medicine should stay true to its core values while adjusting its cultural and operating practices to societal change and confronting facts head-on without losing mission" [59].

The physicians, most commonly through a physician group plan provide the social responsibility, manpower, personal interface on the community and national academic level, and most likely leadership. The physicians, particularly if well organized should have the ability to bring experience from interactions with patients, researchers, and other health care providers to create an environment consistent with the vision that they have helped to create. They provide the leadership for the culture of the organization.

It is well known that problems can arise when any organized group acts outside of its core competencies [46]. For the health care organization to be successful then, there must be a recognition that modern leadership is more collaborative and wields a different kind of authority than in the past [31] and recognition that the delineation of responsibility within the core competencies is vital and part of the internal discipline of the organization. For example, the chief medical officer in the hospital would be accountable for physician performance and quality in the hospital, the dean of the medical college responsible for academic performance, educational initiatives, research and leadership development, and the leader of the physician group accountable for financial performance and outpatient facilities. A council of this leadership would be necessary for effective integration.

A significant amount of effort of all stakeholders should be spent building relationships to achieve the shared vision. Because the core competencies vary among the stakeholders, and because the resource allocation should optimize the output, there should be frequent discussions of the values, purpose, and socially meaningful outputs that are desired. It will be necessary to use national benchmarks [60,61], social responsibility, and market forces to determine some financial relationships.

### A Shared Metric

As mentioned earlier, the output in social organizations, of which an academic health care system is one, should be measured not only in financial terms but in terms of the good it can do for society [28]. In *Good to Great and the Social Sectors: A Monograph to Accompany Good to Great *Jim Collins outlines the difference between measuring an organization's financial performance and its social performance. Although strong finances are obviously required to keep the system operating and functional and money is considered an input, its success is measured on its social output. This might be, for example, a measure of well-conducted survey that measures health related quality of life [62]. It might also be the ability to serve all of the health care needs in a region or word-of-mouth recognition nationally that an institution provides excellence in care. Reducing health disparities is another possible example of a health care organization's output. Regardless of the metric, it should be consistent with the core values, and there should be agreement among all stakeholders that it will be used.

### Summary

The president and CEO of the AAMC, Dr. Darrell Kirch, suggested that the culture of academic medicine needed to change. "While higher education and health care have held fast to their traditional, individualistic culture, that the world has fundamentally changed to a greater emphasis on collaborative, coordinated, and integrative efforts in research, patient care, and medical education"[63]. It frequently becomes obvious when addresses like this are shared that for physicians, medical schools, and hospitals to serve the needs of society in a gratifying way, there will need to be change. There needs to be higher quality and lower cost. There needs to be more scientific and social advances. There needs to be a stronger sense of purpose and a mechanism for sustainability. It is clear that there is a real and urgent need for relationship building among the professionals whose duty it is to provide these services.

## Competing interests

The authors declare that they have no competing interests.

## Authors' contributions

JPK edited, drafted and revised the contents of the manuscript, RO assisted with editing and review of the manuscript. All authors read and approved the final manuscript.

## Pre-publication history

The pre-publication history for this paper can be accessed here:


